# Quality Improvement Targeting Non-pharmacologic Care and As-needed Morphine Improves Outcomes in Neonatal Abstinence Syndrome

**DOI:** 10.1097/pq9.0000000000000612

**Published:** 2022-11-10

**Authors:** Thomas J. McMorrow, Kristen Byrnes, Megan Gates, Tai Hairston, Aysha Jawed, Megan Keydash, Sonya Ulrike Steele, Dörte Thorndike, Liselotte van Londen, Benjamin E. Bodnar

**Affiliations:** From the * Division of Pediatric Emergency Medicine, UPMC Children’s Hospital of Pittsburgh, University of Pittsburgh School of Medicine, Pittsburgh, Pa.; † Department of Pediatrics, Johns Hopkins Children’s Center, Johns Hopkins University School of Medicine, Baltimore, Md.

## Abstract

Despite the rising incidence of neonatal abstinence syndrome (NAS), there remains wide practice variation in its management. Many recent studies have focused on implementing new symptom scoring systems, typically as part of larger improvement interventions. Despite the continued use of the Finnegan Scoring System, we performed a quality improvement project to reduce the day of life at discharge and cumulative opioid exposure for newborns with NAS.

**Methods:**

We developed a protocol for NAS treatment emphasizing early transfer to general pediatric units, maximization of non-pharmacologic care, and use of as-needed morphine whenever pharmacologic treatment is required. Outcome metrics were the day of life at discharge and cumulative morphine exposure. As a process measure, we also monitored the day of life at transfer to general pediatric units. In addition, we utilized statistical process control charts to track changes in performance.

**Results:**

Twenty-eight patients met the inclusion criteria for analysis over 24 months following project initiation. Day of life at discharge decreased by 61% (20.0 versus 7.89 days, *P* < 0.001), and cumulative morphine exposure decreased by 81% (13.66 versus 2.57 mg morphine, *P* ≤ 0.001). Day of life at transfer to general pediatric units decreased by 49% (11.13 versus 5.7 days, *P* = 0.002). There were no readmissions or other identified adverse events.

**Conclusions:**

We achieved significant improvements in NAS outcomes using improved non-pharmacologic care and as-needed morphine. Moreover, the improvement did not require transitioning to a new scoring system. These results support the efficacy and safety of as-needed morphine for NAS management.

## INTRODUCTION

### Problem Description

Opioid use disorder continues to be a public health crisis within the United States. Between 2010 and 2017, pregnant women with opioid-related diagnoses documented at delivery increased by 131%, while babies born with symptoms of opioid withdrawal, known as neonatal abstinence syndrome (NAS) or neonatal opiate withdrawal syndrome, increased by 82%.^1^ Maryland is 1 of only 7 states reporting greater than 27.8 opioid-involved overdose deaths per 100,000 people annually, the highest rate categorized by the National Institute on Drug Abuse.^2^ Consequently, the incidence of NAS in Maryland is more than twice the national average, with 13.7 NAS diagnoses per 1000 newborns in 2018, as compared with the national average of 6.8.^3,4^ Increases in opioid use and overdose during the COVID-19 pandemic may foreshadow an increase in the incidence of NAS nationally and highlight the need to optimize care for these infants.^5–8^

### Available Knowledge

Treatment of NAS varies across care contexts, providers, and institutions. Traditional management often relies upon symptom scoring using a version of the Finnegan Neonatal Abstinence Score (FNAS) with regular use of opiates and adjunctive medications. Hospitalizations often last several weeks, during which providers gradually wean infants from these medications. Many practitioners are concerned that common practice underutilizes non-pharmacologic symptom control measures despite non-pharmacologic therapy having long been recommended as first-line therapy for NAS.^9,10^

Recently, various studies and quality improvement (QI) initiatives have aimed to rethink multiple components of NAS care. This work has shown that caregiver rooming-in and improved non-pharmacologic care are associated with lower rates of pharmacotherapy for withdrawal and decreased length of stay (LOS).^11–13^ There is also growing interest in as-needed opioid therapy, with initial studies showing it may decrease LOS and opiate exposure.^14–16^ Care setting also impacts patient outcomes, with admission to general pediatric units compared with the neonatal intensive care unit (NICU) associated with a decreased LOS and opiate exposure.^17^

Many published QI initiatives give considerable attention to withdrawal assessment tools. The FNAS remains the most used tool, although it has not been subject to rigorous comparative study, and questions remain regarding its validity.^15,18–27^ Recently, a quality improvement team developed the Eat, Sleep, Console tool, and its implementation has been associated with decreased LOS and reduced opiate exposure. However, whether these benefits were attributable to the tool or other QI processes remains unclear.^16,28–30^ Notably, in a recent clinical report, the American Academy of Pediatrics did not endorse one scoring tool over another, citing a lack of evidence demonstrating the superior validity of any one tool.^31^

### Rationale

**Fig. 1.**
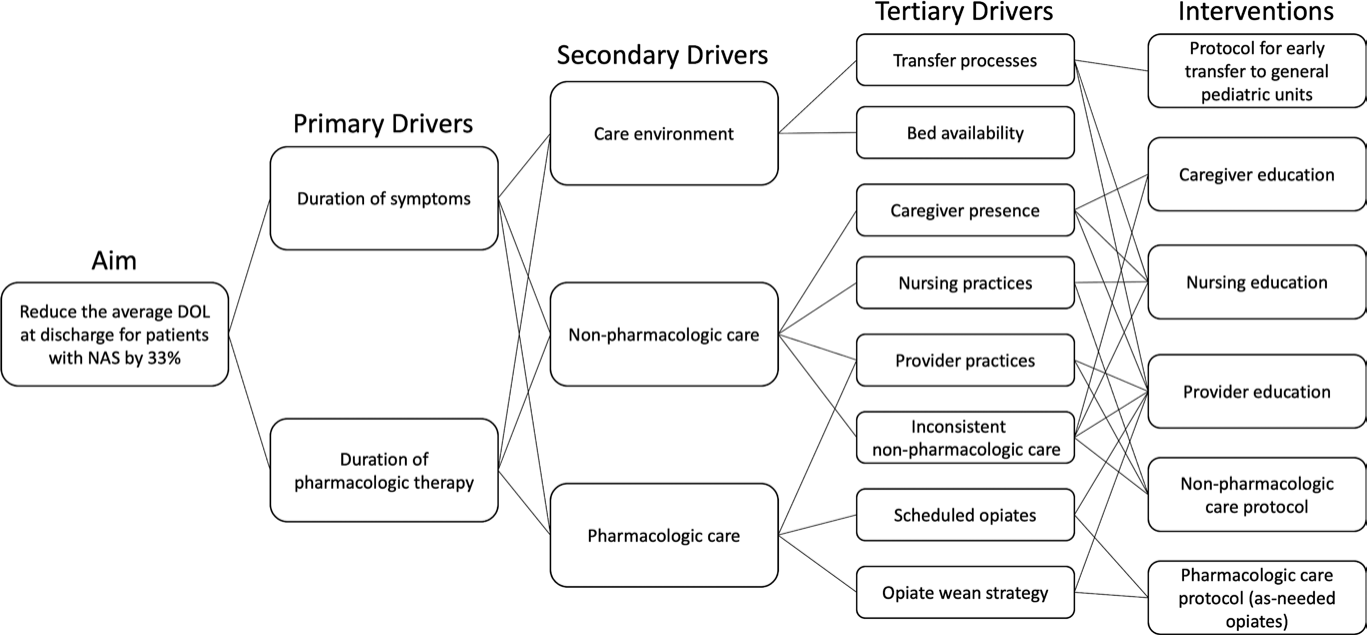
Key driver diagram for the project’s primary outcome measure, the DOL at discharge for patients with NAS, and project interventions targeting these drivers.

Our improvement package included (1) early transfer to general pediatric units, (2) an improved non-pharmacologic care bundle, and (3) a pharmacologic treatment protocol that included the use of as-needed rather than scheduled morphine (Fig. 1).

Based on the known importance of environmental control and rooming-in as components of high-quality non-pharmacologic care,^11–13^ we expected that early transfer to general pediatric units (from the newborn nursery or NICU) would improve these elements of care.

Historically, medical providers have used a variety of non-pharmacologic care interventions for the treatment of NAS. While most experienced caregivers are familiar with these interventions, consistent implementation remains challenging. Therefore, we expected a standardized protocol to support high-quality non-pharmacologic care and coordination among multidisciplinary caregivers to improve the quality of care.

Given the lack of definitive evidence supporting a particular scoring system and the potential burden of implementing a novel system, our project focused on the cautious interpretation of the FNAS score within an integrated clinical assessment of the patient’s status.

We expected improved non-pharmacologic care to decrease but not eliminate the need for pharmacologic treatment. Given the lack of evidence supporting scheduled opiates as a superior approach, we implemented an as-needed dosing approach. We expected an as-needed dosing strategy to significantly reduce the total opiate exposure in opiate-treated infants while still allowing regular dosing if indicated by the clinical picture.

### Specific Aims

Our project aimed to improve the quality of NAS care. Specifically, we aimed to decrease the average day of life (DOL) at discharge for patients with NAS by 33% over the project’s first year. An additional aim was to decrease cumulative morphine exposure. Our primary balancing measure was readmission within 30 days of discharge.

## METHODS

### Context

The Johns Hopkins Children’s Center (JHCC) is a 209-bed tertiary care academic medical center in Baltimore, Maryland. It has approximately 9000 inpatient pediatric admissions annually, with 85% of patients residing in Maryland. On average, 1–2 patients with NAS are treated in general pediatrics units each month, with additional patients receiving care exclusively in the newborn nursery or NICU. Traditional care used the FNAS for symptom scoring, scheduled morphine as first-line opiate therapy, and clonidine as second-line pharmacotherapy.

### Interventions

We assembled a multidisciplinary QI team, including pediatric hospitalists, residents, advanced practice providers, nurses, social workers, and case managers.

The QI team provided early and ongoing education to physicians and nurses on the initiative, emphasizing non-pharmacologic therapies. We developed new guidelines outlining an optimized process for the early transfer of patients with NAS to general pediatric units and distributed them to the newborn nursery and NICU in October of 2019. (**See Supplemental Digital Content**, Guidance on the Transition of Care, http://links.lww.com/PQ9/A423.)

In addition, based on existing literature and the expertise of the QI team, we developed a protocol for the standardized care of infants with NAS. (**See Supplemental Digital Content,** Pedi Floor NAS Guideline, http://links.lww.com/PQ9/A426.) We distributed binders containing the protocol to general pediatric units in January 2020. We updated the protocol iteratively based on ongoing performance reviews and staff feedback. Over time, we also created educational documents for caregivers and staff and integrated them into the protocol. Resources included basic and detailed caregiver education sheets on NAS and our care approach. [**Supplemental Digital Content**, JHCC NAS Parent Information (basic), http://links.lww.com/PQ9/A424; **Supplemental Digital Content**, JHCC NAS Parent Information (detailed), http://links.lww.com/PQ9/A425; **Supplemental Digital Content**, The Cuddler Checklist (a menu of non-pharmacologic approaches), http://links.lww.com/PQ9/A427; **Supplemental Digital Content**, My Comfort Care Plan (a laminated family-staff communication aid that sought to empower caregivers to drive their child’s care), http://links.lww.com/PQ9/A429; and **Supplemental Digital Content,** Unit Huddle Checklist for Patients with NAS, http://links.lww.com/PQ9/A428.]

### Study of the Interventions

We identified patients with NAS cared for on general pediatric units during the project via direct reporting by clinical staff to the QI team. We obtained quantitative and qualitative data by manual chart review. The QI team completed case-based reviews for all cases at least monthly and solicited input from the primary care team whenever possible.

While we applied the updated treatment approach to all patients receiving care in this context, we excluded patient data from analysis if a non-NAS medical condition determined the duration of their hospital course (eg, they required brief NAS treatment but prolonged hospitalization for persistent feeding difficulty). In addition, we included data for infants with unknown or polysubstance exposure.

### Measures

We assessed DOL at discharge as the primary outcome measure and cumulative morphine exposure (in milligrams) as a secondary outcome measure. Furthermore, we monitored DOL at transfer to general pediatric units as a process measure. The primary balancing measure was readmission within 30 days of discharge. We also performed a qualitative review for the presence of acute complications of NAS potentially attributable to the new protocol (such as seizures or the need for escalation to a higher level of care).

### Analysis

We used Minitab (v18-20) statistical process control (SPC) charts with standard definitions of statistical significance to identify special cause variation. First, we reviewed SPC charts and performance trends in real time. Then, we retrospectively compared baseline mean performance with performance during the project using a 2-sample *T*-test (DOL at transfer) and 1-way ANOVA when multiple phases were present (DOL at discharge and cumulative morphine exposure).

### Ethical Considerations

The Johns Hopkins Medicine Institutional Review Board certified this project as a QI activity and waived the requirement for written informed consent (IRB00214884). The QI team reported no conflicts of interest.

## RESULTS

The baseline data period spanned March 2018 to March 2019, during which we identified 16 patients as appropriate for inclusion in the analysis. After project initiation, from July 2019 to June 2021, 33 patients received care for NAS in general pediatric units; we ultimately included 28 patients in the analysis. In addition, we excluded 5 patients because non-NAS medical conditions determined the duration of their hospital course.

**Fig. 2.**
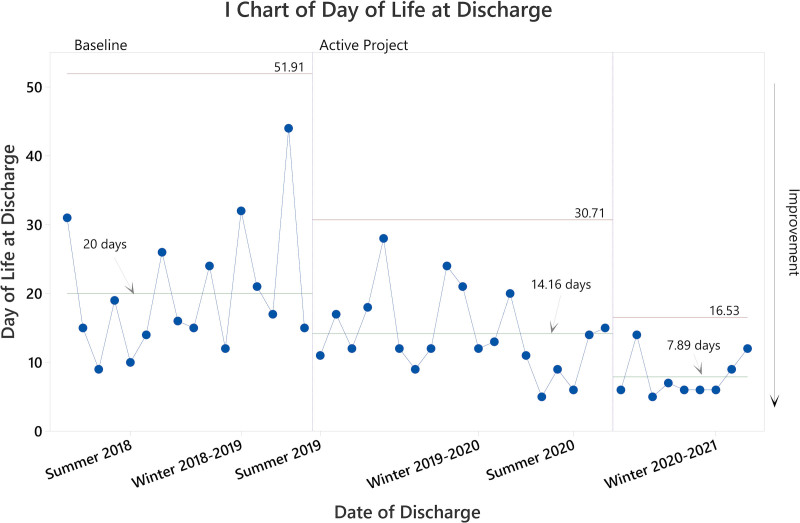
SPC chart demonstrating improvement in the DOL at discharge for patients with NAS at JHCC following project initiation. We represented patients chronologically, moving from left to right. Vertical borders represent statistically distinct performance phases. The first performance phase shift occurred immediately following project initiation. The mean DOL at discharge is shown for each phase.

**Fig. 3.**
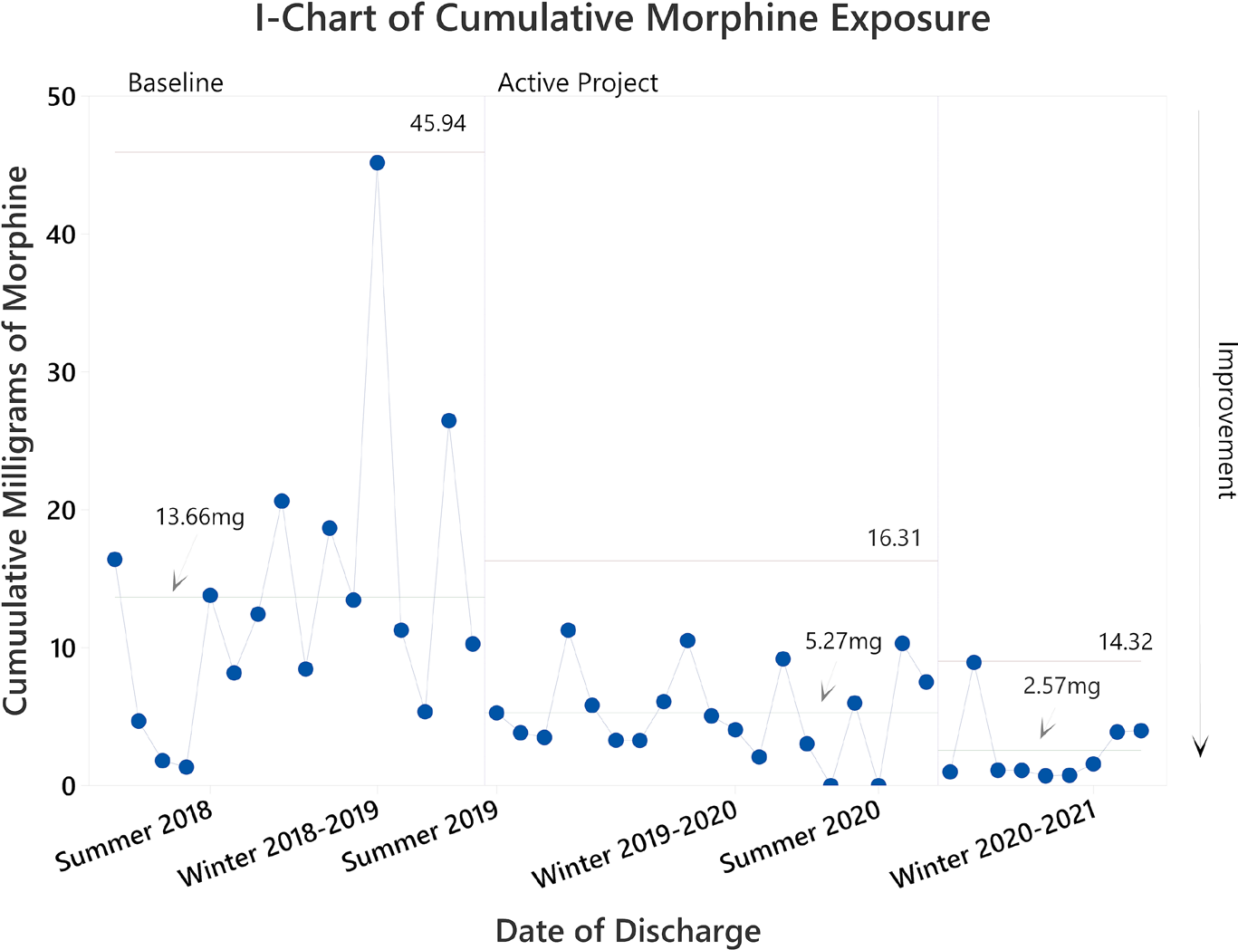
SPC chart demonstrating improvement in cumulative morphine exposure at discharge received by patients with NAS at JHCC following project initiation. We represented patients chronologically, moving from left to right. Vertical borders represent statistically distinct performance phases. The first performance phase shift occurred immediately following project initiation. The mean cumulative morphine exposure in milligrams is shown for each phase.

**Fig. 4.**
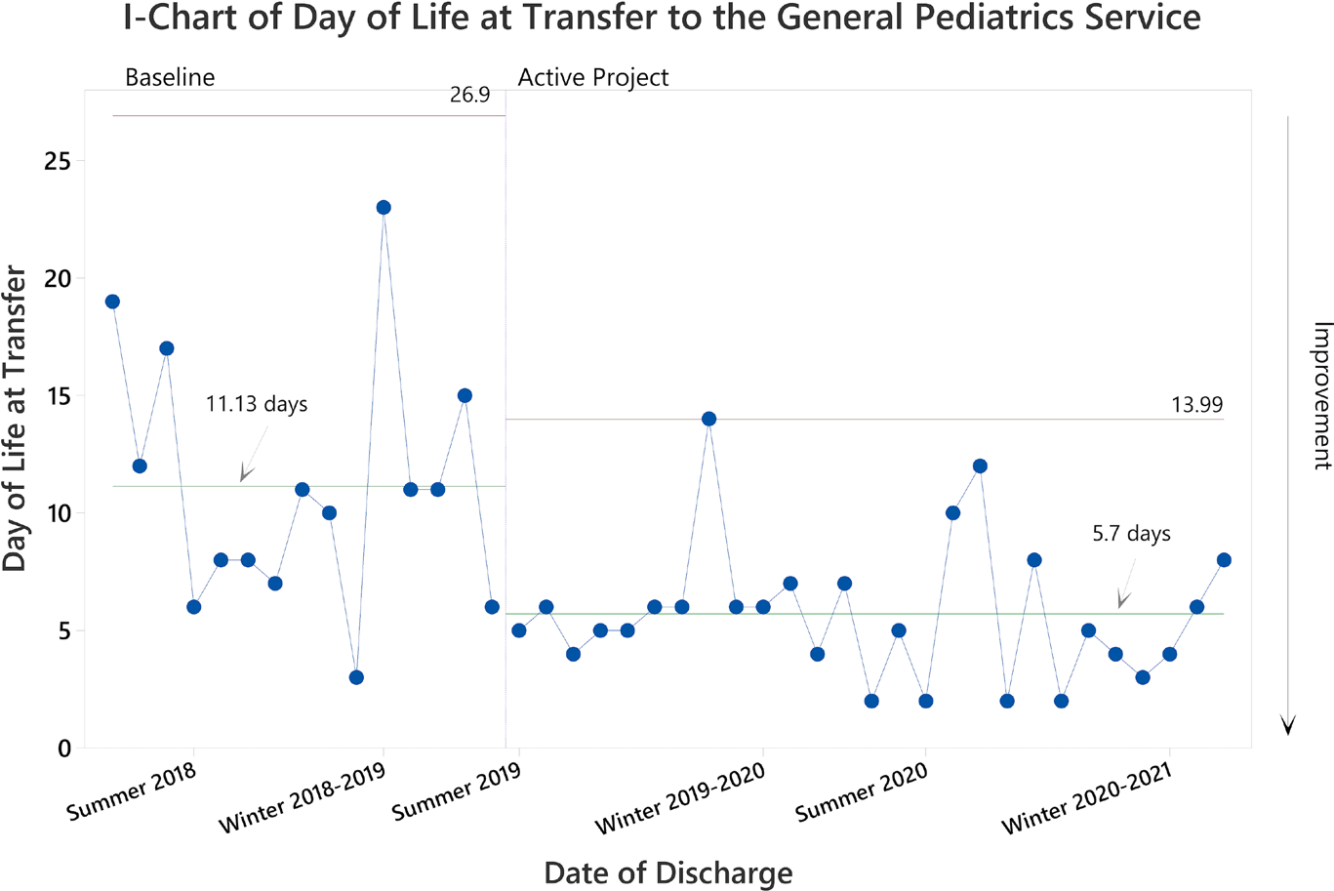
SPC chart demonstrating improvement in DOL at transfer to general pediatric units for patients with NAS at JHCC following project initiation. We represented patients chronologically, moving from left to right. Vertical borders represent statistically distinct performance phases. The first performance phase shift occurred immediately following project initiation. The mean DOL at transfer to general pediatric units is shown for each phase.

In the baseline cohort, the mean DOL at discharge was 20 days (median 16.5 days; Fig. 2); the mean cumulative morphine exposure was 13.66 mg (median 11.86 mg; Fig. 3); and the mean DOL of transfer to the general pediatric units was 11.13 days (median 11; Fig. 4). Providers treated all patients with morphine, and 31% (5 of 16) also received clonidine. In addition, providers transferred 31% of patients (5 of 16) to another medical institution for ongoing care, with 80% of transferred patients (4 of 5) still receiving opiate therapy for NAS. As such, the total opiate exposure is underestimated in this subpopulation.

During the final phase of the project, the DOL at discharge decreased to a mean of 7.89 days (a 61% decrease compared with baseline, *P* < 0.001 by 1-way ANOVA; Fig. 2); the mean cumulative morphine exposure decreased to 2.57 mg (an 81% reduction compared with baseline, *P* ≤ 0.001 by 1-way ANOVA; Fig. 3); and the mean DOL of transfer to general pediatric units decreased to 5.7 days (a 49% decrease compared with baseline, *P* = 0.002 by 2-sample *T*-test; Fig. 4). Ad hoc analysis excluding the notable high outlier in the baseline cohort did not impact statistical findings (*P* < 0.01 for all comparisons).

During our active project, providers treated 26 of 28 patients (93%) with morphine, and 2 of 28 patients received clonidine (7%). All patients were initiated on these pharmacotherapies before transfer to general pediatric units. In addition, providers transferred 1 patient to another medical institution for ongoing care, although the indication was for feeding rehabilitation, and the patient was not receiving opiates at the time of transfer.

We did not identify any adverse events related to project interventions during the initial 2 years of the project. In addition, no patients were readmitted to our hospital system within 30 days of discharge.

It is important to note that most of the initial 2 years of the project occurred during the COVID-19 pandemic. As a result, the pandemic delayed some planned interventions, including plans for a volunteer-based non-pharmacologic care support team (“cuddlers”). In addition, for a significant portion of the project, COVID-related restrictions allowed only 1 caregiver at the bedside, limiting other potential sources of non-pharmacologic care support. However, these restrictions did not prohibit caregivers from entering and exiting the hospital as desired.

Of note, even after implementing our multidisciplinary protocol on general pediatric units, standard practice in the newborn nursery and NICU continued to include scheduled morphine without sustained efforts to change non-pharmacologic care techniques. As a result, patients were routinely transferred to general pediatric units already receiving scheduled opiates. By early in the second year of the project, providers on general pediatric units were consistently transitioning all patients with NAS to as-needed morphine within 1 calendar day of transfer (SDC, P chart of patients transitioned to PRN morphine within 1 day of transfer, http://links.lww.com/PQ9/A437), with most patients self-weaning from opiates with minimal additional pharmacotherapy. Thus, the timing of transfer to general pediatric units remained a major determinant of total opiate exposure. The timing of the transfer was, in turn, frequently impacted by limitations in bed availability, which was beyond the scope of our QI project.

## DISCUSSION

### Summary

Using a package of improved non-pharmacologic therapies, supplemented by as-needed pharmacotherapy, and without abandoning the FNAS system, our project significantly reduced DOL at discharge and cumulative morphine exposure for patients with NAS. We felt the expedited transfer of these patients to general pediatric units to be a key in achieving these outcomes. Despite these changes, there were no documented project-related adverse events or NAS-related readmissions.

The strengths of our study included a reliance on non-pharmacologic care approaches that can be easily reproduced, integration of multidisciplinary education supporting culture change, and pharmacologic approaches that do not require the use of nonmorphine opiates, with which many providers have limited comfort.

Given existing knowledge, it is unsurprising that an improved non-pharmacologic care package improved NAS outcomes. However, it is notable that we obtained these improvements without dedicated focus on the scoring system or transitioning from the FNAS to a new assessment tool. In addition, our findings contribute to the existing literature on the efficacy and safety of as-needed opiate dosing for NAS.

### Interpretations

Project interventions led to sustained improvements in DOL at discharge and cumulative opioid exposure throughout the active study period. It did not appear that any single intervention in the project package was a unique driver of improvement, but rather their combination supported progressive improvement and staff culture change. Nursing and provider culture change regarding the efficacy of non-pharmacologic care was gradual; however, it was crucial for success and supported sustainability.

Although we emphasized caregiver engagement in bedside management as a key component of improved non-pharmacologic care, in the absence of caregivers or other family members, these additional responsibilities largely fell on the nursing staff. This intervention would not have been sustainable without nursing culture change, as non-pharmacologic approaches are often more time-consuming and labor-intensive than pharmacotherapy. As comfort with the care of these patients improved on general pediatric units, widespread staff acceptance of non-pharmacologic approaches contributed to the continued improvements in patient outcomes.

Project interventions occurred largely on the general pediatric units. Although our project continues to plan additional collaboration with the newborn nursery and NICU, the differing care approaches added complexity to care transitions, especially if we did not appropriately educate parents and providers on the rationale for the changes. Focusing on unified treatment approaches across care contexts, and collaborative transitions of care between medical treatment contexts and upon discharge continue to be opportunities for improvement and focus on patient- and family-centered outcomes.

We developed and implemented interventions with relatively limited financial resources, although significant staff time was necessary, especially that of QI team leaders. Therefore, a quantitative analysis of cost savings was beyond the scope of our project. However, it appears likely that the project led to significant reductions in the cost of care for these patients. In addition to shifting care from NICU beds to lower cost general pediatric beds, project interventions directly reduced 220 hospital days during the active study period. This reduction was calculated by multiplying the number of included patients (28) by the mean decrease in DOL at discharge during the entire active study period (7.86 days).

Direct comparison with other published studies is difficult because our intervention package differed from those documented elsewhere. However, our findings are consistent with previous studies that have examined the impact of improved non-pharmacologic care bundles. In addition, while there are few reported specific regimens for as-needed opioid therapy, our results are consistent with the limited published experience, which found it safe and effective.^14–16^ Although clinical experience supports the proposition that decreasing the duration of hospitalization for initial treatment of NAS is safe when done appropriately, there remains a lack of data on the impact of initial treatment approaches (including opioid exposure) on long-term neurodevelopmental outcomes.^32,33^ More studies are needed in these areas.

As our project continues, it will intensify its focus on patient- and parent-centered outcome metrics, including a more direct assessment of parental presence at the bedside and caregiver experience. Given that our project did not focus on novel scoring systems, it is possible that a transition to such a system, or a move away from scoring to holistic assessment, may have additive impacts on outcomes. Moreover, while it is outside the scope of our current project, further evaluation of as-needed dosing regimens, including the use of nonmorphine-based as-needed opiate regimens, is likely to be a worthwhile target for future research or QI initiatives.

### Limitations

We conducted our project at a single tertiary care center with a relatively small sample size. We provided care on general pediatric units with all private rooms and on which ancillary services, such as social work, child life, and case management, were available. A caring environment that allows for a private room and aggressive control of environmental stimuli is important and maybe the main resource limitation for implementation in some contexts. Furthermore, while our outcomes aligned with or exceeded expectations, implementing a novel care approach only on general pediatric units also likely limited project impact. Finally, the fragmentation of NAS care across departments at our institution is not unique and maybe a potential barrier at other institutions.

As a QI project, we cannot reliably isolate the effect of any single component of project interventions on NAS outcomes. Our small sample size precluded advanced analysis, such as via interrupted time series. Additional ad hoc analysis eliminating high baseline outliers and considering mean and median data points did not alter our findings. Thus, it appears unlikely that these changes would have occurred without the project interventions.

Finally, implicit or explicit bias toward parents with opiate use disorder remains highly prevalent. Addressing these biases is difficult but important in supporting further improvement in caregiver engagement in non- pharmacologic care approaches.

## CONCLUSIONS

This project builds on existing knowledge about the safety and effectiveness of non-pharmacologic care in NAS treatment. Significant improvements in care are possible even if practical considerations limit the implementation of novel scoring approaches, which have been the focus of much of the recent literature. Our approach has the potential to be replicated across institutions without the need for significant commitment of resources or advanced staff training. The results of our project further support the use of as-needed rather than scheduled opiates for the pharmacotherapy of infants with NAS.

## DISCLOSURE

The authors have no financial interest to declare in relation to the content of this article.

## Supplementary Material


