# Supplementary material for: Quality Improvement Targeting Non-pharmacologic Care and As-needed Morphine Improves Outcomes in Neonatal Abstinence Syndrome
Source: Pediatr Qual Saf. 2022 Nov 10;7(6):e612. doi: 10.1097/pq9.0000000000000612 (PMC9649270; doi:10.1097/pq9.0000000000000612)
Supplement: Supplementary file 1 [file pqs-7-e612-s001.pdf]

SDC, Guidance on the Transition of Care

NEONATAL ABSTINENCE SYNDROME AT JOHNS HOPKINS CHILDREN'S CENTER

October 2021

Goal: Transfer patients with NAS from the Newborn Nursery to the Pediatrics Unit(s) should be organized to avoid any period in which a private low-stimulation environment is not available to the patient, and to avoid any period in which around-the-clock parental bedside presence is not possible due to lack of parental accommodations.

Guidance:

- NAS patients *should* be transferred to the Pediatrics Units at any time or age, if the transfer helps to ensure the availability of a low stimulation environment and allows the parents or another caretaker to maximize time at the bedside providing supportive care.
  - NAS patients may be transferred whether they are receiving pharmacologic treatment, non-pharmacologic treatment only, or are only under observation for development of symptoms.
  - Unnecessary transfer of care without clear benefit to the patient should be avoided (such as if the patient is very low risk of developing symptoms, is imminently approaching discharge, or if transfer from the post-natal unit would separate the patient from his or her caretakers).
- Routine neonatal screening and services should be completed prior to transfer to the floor, including congenital heart disease screening, hearing screening, obtaining the first state screening sample, and circumcision when applicable.
- The sending provider from the newborn nursery should notify the shift coordinator of the expected transfer in advance of the time of maternal discharge, although the patient should NOT be transferred prior to maternal discharge.
  - **The sending provider should notify shift coordinators before midnight on the day prior to anticipated maternal discharge** so that the patient may be triaged in advance.
  - When notifying of an expected transfer in advance, notify the bed and shift coordinators of the expected timing of maternal discharge, to aid in planning and triage decisions
  - There is no special triage process for NAS patients, and unit and shift coordinators will need to triage these patients amongst all existing bed requests, which is why advanced notification is important. The timing of transfer cannot be guaranteed.
  - If a patient is younger than 36 weeks adjusted gestational age, or weighs less than 2kg at the time of potential transfer, the sending provider must discuss the case with an accepting floor provider prior to initiating the transfer
- Whenever possible, the mother or another caretaker should be medically ready and otherwise prepared to move with the patient to the Pediatric Unit at the time of transfer.
- Social workers in the Nursery/L&D and the accepting unit should be in contact prior to transfer to discuss any issues with the mother-baby dyad (i.e., custody, potential transfer to rehabilitation facilities [such as Mount Washington]).

Background:
Treatment of Neonatal Abstinence Syndrome (NAS) is a multidisciplinary process, requiring coordination between hospital units, multidisciplinary staff, as well as family and other caretakers. High quality NAS care starts with non-pharmacological and environmental modifications to control symptoms, including minimizing environmental stimuli via a dark quiet environment, responding early to an infant's signals using positioning and comforting techniques, and frequent small volume feeds.[1] Studies have shown that maximizing parental involvement in care, including rooming-in with the infant have significant positive impacts on the intensity of symptoms, the need for pharmacologic treatment, and the duration of treatment and hospitalization.[2,3] Many of these approaches become difficult or impossible after maternal discharge from the post-partum unit, if the physical space to provide a low-stimulation environment and allow for family-centered care becomes unavailable.

References:

1. Hudak ML, Tan RC. Neonatal drug withdrawal. Pediatrics. 2012;129(2):e560. https://www.ncbi.nlm.nih.gov/pubmed/22291123. doi: 10.1542/peds.2011-3212.

2. Holmes AV, Atwood EC, Whalen B, et al. Rooming-in to treat neonatal abstinence syndrome: Improved family-centered care at lower cost. Pediatrics. 2016;137(6):e20152929. https://www.ncbi.nlm.nih.gov/pubmed/27194629. doi: 10.1542/peds.2015-2929.

3. Howard MB, Schiff DM, Penwill N, et al. Impact of parental presence at infants' bedside on neonatal abstinence syndrome. Hospital pediatrics. 2017;7(2):63. https://www.ncbi.nlm.nih.gov/pubmed/28137920. doi: 10.1542/hpeds.2016-0147.
