# Supplementary material for: Quality Improvement Targeting Non-pharmacologic Care and As-needed Morphine Improves Outcomes in Neonatal Abstinence Syndrome
Source: Pediatr Qual Saf. 2022 Nov 10;7(6):e612. doi: 10.1097/pq9.0000000000000612 (PMC9649270; doi:10.1097/pq9.0000000000000612)
Supplement: Supplementary file 2 [file pqs-7-e612-s002.pdf]

SDC, Pedi Floor NAS Guideline – Version 3-3-22

Note on **Shared Responsibility**: When appropriate, individual steps below indicate "Primary:…" and "Secondary:…" responsibility. The expectation is that this step is usually organized or performed by the primary staff member indicated, but it may also be performed by the Secondary staff member(s) when appropriate.

Note on **Transfer on Pharmacologic Therapy**: The sections of this guideline are written to address care from the immediate post-natal period through onset and treatment of NAS, though patients may arrive to the unit already on pharmacologic therapy. This is addressed in Notes at the beginning of each section. Regardless of care prior to transfer, all elements of the care plan on transfer should be reassessed in line with known best practices for the general care unit.

1. **On Arrival to the Unit – Initial Care**
   Note: If patient arrives on the unit already on pharmacologic therapy, *the steps in Section 1 should still be performed if possible.*
   1.1. **Meet with parents** to discuss the importance of Rooming-In
      1.1.1. If parent not present, contact and schedule in-person meeting ASAP [Primary: Physician, Secondary: Nursing]
      1.1.2. Identify barriers to Rooming-In and problem solve solutions [Primary: Physician, Secondary: Nursing, Social Work].
      1.1.3. Consult Social Work, Case Management, Child Life and the Substance Use Disorders Service as needed to provide support and address barriers [Primary: Nursing except physicians will primarily consult the Substance Abuse Disorder Service, Secondary: Physician]
      1.1.4. Complete the "Barriers to Rooming In" Checklist. Formulate plan and mobilize resources to address these barriers (MTA passes, meal tickets, etc., may be available via Social Work) [Primary: Social Work, Secondary: Physician, Nursing]
   1.2. **Provide (or Review) parental education** on NAS Care
      1.2.1. Use the NAS teaching plan in Epic, including MyChart Bedside pre-educational Survey (once available) [Primary: Nursing]
      1.2.2. Additional resources are available: NAS Parent Info "The Basics" and/or "The Details", "The Cuddler Checklist", "My Comfort Care Plan" [Primary: Nursing, Secondary: Physicians, Child Life]
   1.3. **Identify expected gaps in parental presence or Rooming In**
      1.3.1. Document specific times of expected caretaker absence ahead of time whenever possible [Primary: Nursing, Secondary: Physician, Child Life]
      1.3.2. Inform Child Life/Volunteer Coordinator of expected absences to form a plan [Primary: Nursing, Secondary: Physician, Child life, Social Work]
      1.3.3. Child Life/Volunteer Coordinator to contact on call Volunteer pool to attempt to meet scheduled needs as well as respite needs even if parents are rooming in around the clock [Primary: Child Life]
   1.4. **Nursing** will begin **NAS symptom monitoring** on arrival [Primary: Nursing, who should discuss any needed documentation/order changes with Physicians]

- 1.4.1. Scoring should be done AFTER providing non-pharmacologic care including feeding, swaddling and other calming techniques
- 1.4.2. Scoring should be done roughly every 3-4 hours, but longer intervals are acceptable if infant is calm and scoring would disturb the infant
- 1.4.3. Vital Signs should be batched with other care and assessments rather than on a hard schedule unless there is clinical concern for instability. Vital Signs every 8 hours is likely appropriate for most NAS patients.
- 1.4.4. Infant should NOT be awoken or disturbed to assess symptoms if there is no other indication to disturb the infant, regardless of time since last score
- 1.4.5. Components of the score that require disturbing the infant, such as "tremor when disturbed" and "moro reflex" should not be elicited intentionally even when scoring, though may be recorded if observed coincidentally. They may be scored as 0 if not observed.

1.5. Provide and begin to fill out "My Comfort Care Plan" Individualized treatment form [Primary: Nursing, Secondary: Physician, Child life]

1.6. Begin implementation of "The Cuddler Checklist" [Primary: Nursing, Secondary: Child Life, Physician]

1.7. The NAS Unit Huddle and Discharge Checklist should be used at Unit Huddle to ensure resources are appropriately mobilized. [Primary: Nursing Team Leader]

1.8. **Inpatient observation through DOL 5 is reasonable for infants exposed to long acting opiates (buprenorphine, methadone), although those exposed only to short acting opiates (oxycodone, morphine, heroin, fentanyl) may only need to be observed for 72 hours.** In the vast majority of cases, infants exposed to short acting opiates who will develop NAS symptoms within 48-72 hours of birth, although those exposed to long acting medications may not develop NAS symptoms until up to 5 days after birth. [Primary: Physician, Secondary: Nursing]

2. <u>Infant showing early signs of NAS</u>

Note: if patient arrives on the unit already on pharmacologic therapy, Step 2.3 is still required, though other elements of Part 2 are also addressed in Part 1 and 3.

2.1. Providers should **schedule a face to face family education session ASAP** [Primary: Physician, Secondary: Nursing, Child Life though a minimum of physician and nursing should be present at session whenever possible]

- 2.1.1. Discuss developing NAS, address questions and concerns [Primary: Physician, Secondary: Nursing]
- 2.1.2. **Review parental role, Rooming In, and formulate a plan to enlist additional family members** or caretakers (including volunteers) [Primary: Physician and Nursing, Secondary: Child Life]
- 2.1.3. Identify barriers, problem solve solutions, and provide parental supports (as in Phase 1 – sections 1.1.2 through 1.1.4)
- 2.1.4. **Review The Cuddler Checklist with family and teach techniques** [Primary: Nursing, Secondary: Child Life, Physician]

2.2. Update "My Comfort Care Plan" Individualized treatment form and share with team [Primary: Nursing, Secondary: Physician, Child Life]

2.3. Establish plan for maximization of non-pharmacologic supportive care [Primary: Physician to ensure plan identified, though Nursing, Child Life, Social Work may have primary role in determining details of plan]

3. <u>Infant with progressive or severe NAS and Ongoing Care</u>

Note: if patient arrives to the unit already on pharmacologic therapy, all elements of care should be reassessed in order to maximize non-pharmacologic care, and transition to best practices for pharmacologic care, as below.

3.1. **If patient is developing symptoms of severe NAS then nurse or provider should Initiate a Bedside Huddle** within 30 minutes to include Nursing and Provider (at a minimum), plus Parent (whenever possible, although should not be delayed due to lack of family member) and Child Life (whenever available). This should be done regardless of symptom score if there is clinical concern, though some use a threshold such as Modified Finnegan score >8 on consecutive scores >1 hour apart, or a single score greater than 10 **despite non-pharmacologic care** [Primary: Nursing, Secondary: Physician, Child Life]

3.1.1. Review "The Cuddler Checklist" with emphasis on Rooming-In, and filling gaps in Rooming-In and supportive care [Primary: All present at Bedside Huddle]

3.1.2. Review and update "My Comfort Care Plan" to ensure individualization of care plan [Primary: All present at Bedside Huddle]

3.1.3. **Create a concrete plan for (a) additional non-pharmacologic interventions and (b) re-examination and reassessment of plan** (e.g. nurse will provide comfort care for 10 minutes until volunteer available, team will pursue X,Y,Z environmental and comfort measures, and physician will re-examine in roughly 60 minutes, after next attempted diaper change, feed and comfort measures) [Primary: Physician, Secondary: Nursing, Child Life]

3.1.4. <u>The Bedside Huddle is intended to be used primarily with the onset of severe NAS to unify the care plan across the multidisciplinary team. It is not intended to be used every time elevated scores are documented, although it would be appropriate to repeat a huddle if there is a notable change in clinical status at any time, or to establish a multidisciplinary plan at the time of a transfer.</u>

3.2. **There is no set score for initiation or continuation of pharmacologic opiate therapy.** If an infant continues to have progressive or persistent symptoms of NAS, which impair newborn function or create risk of significant morbidity, despite maximization of non-pharmacologic care, then addition of pharmacologic treatment is reasonable [Primary: Physician (with input from nursing and caretakers)].

3.2.1. Initiation or continuation of pharmacologic therapy is NOT an indication for decreased non-pharmacologic care, rather, it is an indication that further intensification is needed. Ongoing updates to "My Comfort Care Plan", review of educational materials with family and use of "The Cuddler Checklist" with hands-on support/teaching, and maximization of Volunteer services are required. [Primary: Multidisciplinary team]

3.2.2. Initial recommended dosing of Morphine is 0.04mg q3 PRN "severe NAS symptoms unresponsive to non-pharmacologic techniques" [Primary:

Physician, Secondary: Nursing (may contact physician regarding order format/details)]

3.2.2.1. **The multidisciplinary team should determine on an individualized basis what symptoms constitute an indication for PRN dosing**. Assessment of the overall clinical and functional status is the preferred approach to determining the need for pharmacotherapy, rather than strict adherence to numerical scoring. The trend and responsiveness of NAS symptom scores to non-pharmacologic care may be considered as part of this assessment. [Primary: shared decision making between Physician and Nursing, Secondary: Child Life]

3.2.2.2. Indications for PRN dosing may be documented in an updated PRN order, a "Nursing Communication" order and/or discussed between the provider and nursing teams directly. Dosing indications may change over the course of treatment. [Primary: Physician, Secondary: Nursing (may contact physicians regarding presence/absence/format of order)]

3.2.3. **Uptitration of morphine** dosing may be done in increments of 0.02 to 0.04mg per dose, depending on clinical response [Primary: Physician, Secondary: Nursing]

3.2.4. **An "extra" one-time morphine dose may be appropriate** for treatment of breakthrough symptoms without increasing the regular q3 PRN order [Primary: Physician, Secondary: Nursing]

3.2.5. **Morphine dosing should be kept PRN throughout the treatment course even if the dose is escalated. On receipt of a transfer it is recommended to change around-the-clock orders to PRN orders at the same dose and frequency.** While morphine may still be given at 3-hour intervals if needed, there is no need for a standing order [Primary: Physician, Secondary: Nursing]

3.2.6. **There is no data to support any particular opiate weaning regimens, and progress should be based upon clinical response.** Most infants receiving PRN dosing will wean automatically by decreasing dose frequency. In addition, **weaning of both dose and interval may be considered.** Note: Morphine half-life ranges from 4.5 to 13.3 hours in neonates, and may be up to 20 hours in preterm infants [Primary: Physician, Secondary: Nursing]

3.3. Clonidine is the adjunctive medication of choice in cases of severe opiate-induced NAS not controlled with escalating morphine doses ≥0.2mg per dose [Primary: Physician, Secondary: Nursing]

3.3.1. Initial dosing is 0.5mcg/kg/dose q3 hours, which may be escalated to 1mcg/kg/dose q3 hours depending on response. Discuss with a pharmacist or expert if higher dosing is under consideration (it is sometimes used).

3.3.2. Clonidine should be continued at maximum dose until after weaning of opiates, and then may be weaned in half for roughly one day, then off. Longer weans may be needed if higher peak doses are used.

3.4. Use of phenobarbital is not routinely recommended, though may be considered in cases where there is concern for significant benzodiazepine withdrawal. Use should be discussed with an expert [Primary: Physician, Secondary: Nursing]

3.5. **In general, discharge may be considered after a patient has remained stable for 24-48 hours after the last opiate or adjuvant medication dose.** The

observation period may be extended or shortened on a case-by-case basis, based on the risk of symptom recurrence in the infant, and considering the level of non-pharmacologic care available in the post-discharge context. [Primary: Physician, Secondary: Nursing]

3.5.1. **In patients rapidly weaned off high dose opiates a longer period of observation should be considered.**

3.5.2. **If not discharging to home, the capacity of the post-discharge destination to provide non-pharmacologic care should be considered when determining the duration of observation.** For instance, many sub-acute hospitals limit parental presence at the bedside, do not have private rooms, and have higher patient:nursing ratios which may limit the provision of non-pharmacologic care and present as recurrence of symptoms after transfer.

3.5.3. Use of the following **Discharge Checklist** is recommended (adapted from The AAP Clinical Report on NOWS (10-2020):

[ ] No significant signs of withdrawal (off medication) for 24-48 hours, other than those which can be managed by the caretaker outside the hospital
[ ] No other indication for ongoing hospitalization (feeding, comorbidity, etc.)
[ ] Safe discharge care plan established, coordinated with CPS if needed
[ ] Parent/caretaker education on NAS and routine care emphasizing safe sleep
[ ] Primary care follow-up within 48 hours of discharge
[ ] Referred to early intervention services
[ ] Home nursing visitation referral (if needed)
[ ] Hepatitis C testing follow-up plan in exposed infants (if needed)
[ ] Developmental/behavioral pediatrician referral placed (if needed)

3.6. Transition to a higher level of care - there is no specific NAS-related indication for upgrade to the ICU setting and the vast majority will be managed appropriately on the floor. However, severe NAS can rarely be associated with fever, tachypnea, tachycardia, and seizures amongst other manifestations. **Assessment for Non-NAS causes of instability should always be undertaken if the patient has significant or unexpected clinical worsening.** The decision regarding transfer to the ICU (NICU or PICU depending on availability) in this setting should be determined on a case-by-case basis [Primary: Physician, Secondary: Nursing]
