# Supplementary material for: Quality Improvement Targeting Non-pharmacologic Care and As-needed Morphine Improves Outcomes in Neonatal Abstinence Syndrome
Source: Pediatr Qual Saf. 2022 Nov 10;7(6):e612. doi: 10.1097/pq9.0000000000000612 (PMC9649270; doi:10.1097/pq9.0000000000000612)
Supplement: Supplementary file 3 [file pqs-7-e612-s003.pdf]

SDC, JHCC NAS Parent Information (basic) Version 3-3-22
N.A.S. - Neonatal Abstinence Syndrome. YOU are the best Medicine.

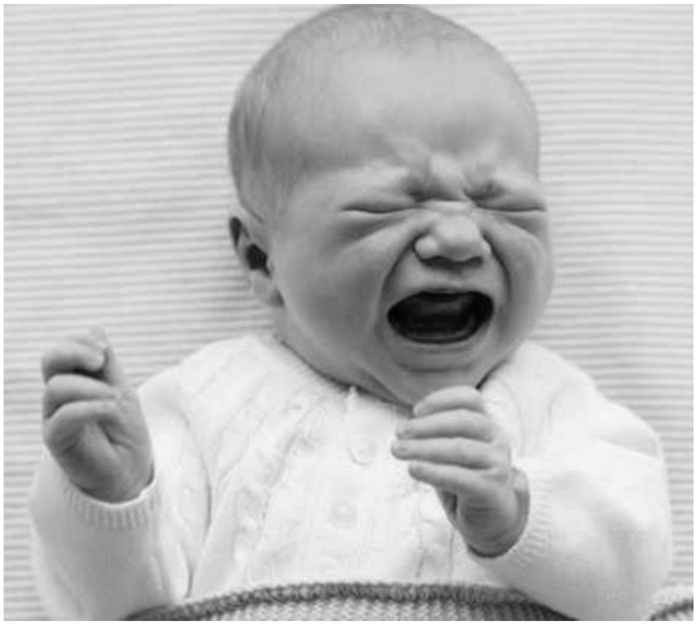

"What is NAS?"

NAS, or Neonatal Abstinence Syndrome, happens when a baby suffers from withdrawal after birth. Your baby is at risk for NAS. If your baby does develop symptoms, YOU are the best treatment.

"What are common symptoms of NAS?"

Babies with NAS tend to be very fussy. They get upset easily and often have symptoms like shakiness or difficulty eating and sleeping.

"What will happen during our hospital stay?"

During your baby's time in the hospital, YOU will be his or her main caretaker. Our team will be watching closely and are here to help. Our team will be watching for sign of NAS for up to 5-7 days, and if NAS develops you and your baby will be in hospital for longer than 5 days.

"How can I help my baby?"

Being with your baby is the best way to help your baby. Babies whose parents stay with them in the hospital often have less severe symptoms. To help calm a fussy baby we recommend:

- <u>Take care of yourself</u> – you need to be healthy for your baby to be healthy. We want to help!
- <u>Always sleep in the same room with your baby:</u> Safe Sleep is still required (talk to your nurse about this)
- <u>Keep a quiet, calm room with dim lighting, even during the day:</u> this may mean limiting visitors also
- <u>Spend as much time "skin-to-skin" as possible</u>: your nurse can show you how if this is new for you
- Use The 4 S's: <u>Swaddling, Shushing (Sound), Swaying, and Sucking</u> (frequent feeding, or pacifier use)
- Prevent skin breakdown: <u>Use butt creams on the diaper area</u> starting right after birth.

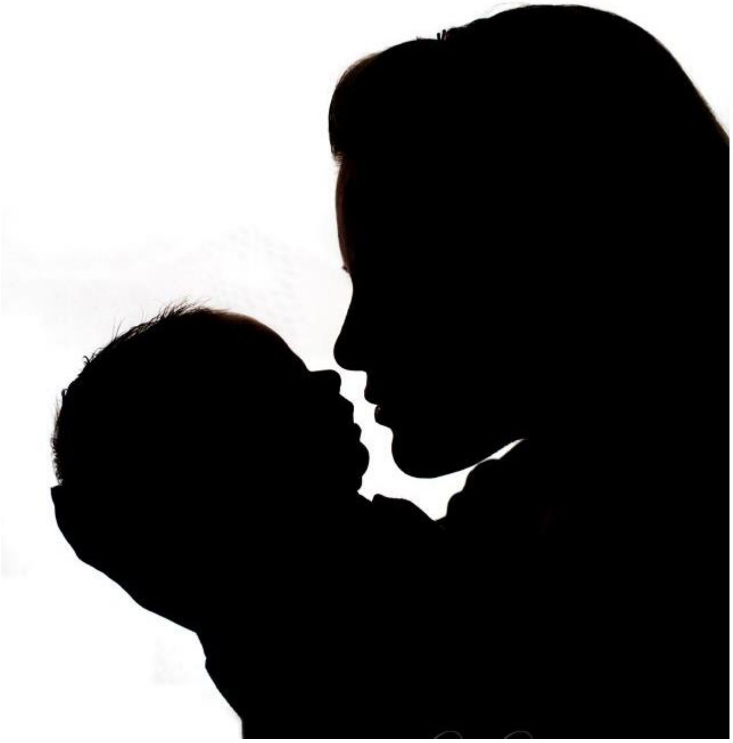

"What happens if my baby does need medicine to treat NAS?"

Some babies need medication like morphine to treat NAS, and <u>that is okay.</u> Even if medication is started, YOU are still the best treatment for your baby, and babies whose parents or family stay with them in the hospital usually get better more quickly. We want to help you make a plan so that you, another family member or a trusted friend is always in the hospital with the baby. Let us know how we can help.

"When can I take my baby home?"

Withdrawal symptoms from NAS improve on their own, whether or not medication is used. Your baby will be ready to go home when any remaining NAS symptoms can be controlled at home, they are feeding and sleeping well, are off any medications for NAS, and there are no other medical issues.
