# Supplementary material for: Quality Improvement Targeting Non-pharmacologic Care and As-needed Morphine Improves Outcomes in Neonatal Abstinence Syndrome
Source: Pediatr Qual Saf. 2022 Nov 10;7(6):e612. doi: 10.1097/pq9.0000000000000612 (PMC9649270; doi:10.1097/pq9.0000000000000612)
Supplement: Supplementary file 4 [file pqs-7-e612-s004.pdf]

N.A.S. - Neonatal Abstinence Syndrome. YOU are the best Medicine.

"What is NAS?"

NAS, or Neonatal Abstinence Syndrome, happens when a baby suffers from withdrawal from medications or other substances after birth. Your baby is at risk for NAS. Not every baby develops withdrawal symptoms, but if they do, they usually show signs by day 5 of life. NAS affects each baby in different ways. If your baby does develop symptoms, YOU are the best treatment.

"What are common symptoms of NAS?"

- Hard to calm down
- Problems eating or sleeping
- Tight muscles in arms and legs
- Trembling, shaking of arms and legs
- Diarrhea
- Sweating, sneezing, fever, or yawning

"What will happen during our hospital stay?"

During your baby's time in the hospital, you will be his or her main caretaker. Our team will be watching closely and are here to help, but your baby will do best when you are around.

- Our team will be watching closely for signs of NAS for up to 5-7 days. If your baby develops serious symptoms, he or she will likely require a longer hospital stay.
- If we are concerned that your baby may be withdrawing, we will teach you ways to help. Always remember - YOU are the best treatment. Sometimes medications are also needed.

"How can I help my baby?"

Being with your baby is the best way to help your baby. Every baby is different and you will have to learn what works best for your baby. We recommend:

- <u>Take care of yourself</u> – you need to be healthy for your baby to be healthy. We want to help you to deal with any issues that may keep you from being present for your baby. Help may be available through our Social Work team, and your doctors, and nurses may be able to help as well.
- <u>Always sleep in the same room with your baby:</u> Keep your baby close to you at all times, so that you can respond quickly to them. But safe sleep habits are still important (talk to your nurse about what this means). Your baby will feel safest and most comfortable with you in the room.
- <u>Calm Environment:</u> Try to keep the room quiet with dim lighting even during the day. Loud noises and bright lights may upset your baby. Limit visitors to not over-stimulate your baby.

- Skin-to-skin care: When you are awake, spend as much time "skin-to-skin" as possible – this means holding your baby against your chest with no layers of clothes between your skin. This helps to calm your baby. If you are breastfeeding, skin-to-skin also helps to promote milk supply.
- Use "The Four S's" to calm your baby: Swaddling, shushing (Sound), Swaying, and Sucking
    - Swaddling – wrap your baby in a light blanket.
    - Sound – use a "sshhhh" sound or white noise machine.
    - Swaying/Motion – try gentle rhythmic rocking or swaying while standing.
    - Sucking/Feeding – feed whenever your baby is hungry and it may be helpful to offer them a pacifier between feedings.
- Prevent skin breakdown: Use butt creams on the diaper area right after birth. Watch elbows and chin closely for redness or irritation from rubbing.

"What happens if my baby does need medicine to treat NAS?"

Some babies need medication to treat NAS, and that is okay. If that's the case for your baby, then your baby will stay in the hospital longer and that is also okay. Even if medication is started, YOU are still the best treatment for your baby, and babies whose parents or families stay with them in the hospital usually get better more quickly. We recommend that you plan ahead so that you or another caretaker are able to stay with your baby at the hospital.

- Plan to have someone watch your other children and/or pets while you are here.
- Plan to have a family member or friend here with you to help you care for your baby.
- If you need to leave for medical appointments or anything else, whenever possible try your best to have a family-member or another person stay and care for your baby with our staff.
- If it is hard for you to talk with your family and/or friends about why your baby is in the hospital, we can help.

"When can I take my baby home?"

We will need to watch your baby closely for signs of NAS for up to 4 to 5 days in the hospital and longer if your baby needs more treatment. Here are some of the main things that will help us decide when your baby will be ready to come home:

- Feeding and sleeping well
- Can be calmed or soothed in ways that can be continued at home
- Gaining weight (or not losing too much weight)
- Temperature, heart rate and breathing are all normal
- Not taking any medications for treating serious symptoms
- Scheduled for a follow-up appointment with a pediatrician and sometimes a homecare nurse
