# Supplementary material for: Quality Improvement Targeting Non-pharmacologic Care and As-needed Morphine Improves Outcomes in Neonatal Abstinence Syndrome
Source: Pediatr Qual Saf. 2022 Nov 10;7(6):e612. doi: 10.1097/pq9.0000000000000612 (PMC9649270; doi:10.1097/pq9.0000000000000612)
Supplement: Supplementary file 5 [file pqs-7-e612-s005.pdf]

SDC, The Cuddler Checklist

Guide to Non-Pharmacologic Care for Neonatal Abstinence Syndrome (NAS)

Note: **Each infant with NAS is different.** Because all the below treatments are low risk, they can be tried on every infant, but **a particular intervention may be helpful, neutral or even agitating for a specific infant**. Use the **My Comfort Care Plan** sheet to document what works and does not work for each patient. Trial and Error is required to find what works best.

ROOMING IN – THIS IS THE MOST IMPORTANT INTERVENTION!

☐ Mother or family member sleeping in the hospital with the baby
☐ Additional Family or other caregiver identified for periods of absence of primary caretaker
☐ Expected absences of primary caregiver identified/documented (such as daily dosing of methadone, physicians visits, etc.)
☐ Plan for team or volunteer presence for planned absence of primary and backup caregiver
☐ Social Work consult completed, discussed provision of supportive resources (MTA passes, meal tickets, etc)
☐ Substance Abuse Disorder Service Consult placed if needed

ENVIRONMENTAL

☐ Lights dim or off
☐ TV off
☐ Phones off or on silent
☐ Curtains closed
☐ Door closed to minimize noise if does not need to be open for observation
☐ White noise machine IF patient not overly sensitive to noise
☐ Limit visitors to 1 in addition to caregiver whenever reasonable, limit visits from children unless they can remain quiet/avoid disturbing infant
☐ Place sign on door – Avoid loud knocks, and minimize interruptions. Check with nurse before entering.

ROUTINE CARE

<u>Feeding:</u>

☐ Small volume, frequent feeds
☐ Immediate responsiveness/feed on demand
☐ Paced feeding or slow flow nipple if difficulty or leakage around mouth
☐ During feed minimize extra noise, uncomfortable touch, visual stimulation (eye contact/faces)
☐ Use a nursing cover or blanket to decrease stimulation with feeds (even if bottle feeding)
☐ Gentle Burping – may be sensitive to touch

Diapering:
□ Immediate responsiveness/change on demand
□ Use diaper cream/ointment with every change
Sleep:
□ Do not disturb during sleep
□ Perform needed care (diaper, feed, change) when already awake or fussy
□ Expect slower than normal transitions from awake or fussy to drowsy or asleep
Touch Sensitivity:
□ Dressing and swaddling which is comfortable and avoids overheating (trial and error)
□ Minimize points of contact from wires/monitors
□ Cautious handling – infants may be sensitive to certain types or locations of touch
Visual Sensitivity (see Environmental section above, plus):
□ Avoid bright colors in visual fields (mobiles, toys, TV)
□ Avoid close face to face and eye contact (except parents/familiar caregiver)
Auditory Sensitivity (see Environmental section above, plus):
□ Speak in soft tones
□ Maximize time hearing parents voice if comforting (unfamiliar voices may be agitating)

EXTRA COMFORT TECHNIQUES FOR WHEN BABY IS GETTING FUSSY

□ Shhh-ing or shussshing noises
□ Swaddling – snug, both arms inside, consider hands to chest so baby can access hands for soothing
□ Pacifier use
□ Breastfeeding if medically safe
□ Skin to skin time
□ Gentle or firm patting on the back – Caution: babies sensitive to touch may NOT like this
Movement/positioning:
□ Rhythmic, non-random movements
□ Head to toe in rocking chair or gentle swing
□ Side to side swaying (head to toe for baby)
□ Vertical rocking (slow up and down movements while infant held upright)
□ Avoid swinging/rotational movements – this is agitating for many babies
□ No shaking or jiggling – vibration made symptoms worse in one study
□ Lying to sitting – move from lying to sitting upright can distract and calm sometimes
□ C-position side-lying – arms and hands folded in front of chest, knees to chest. Infant should NOT be left side-lying unobserved
□ C-Position upright or when held – see above
