# Supplementary material for: Quality Improvement Targeting Non-pharmacologic Care and As-needed Morphine Improves Outcomes in Neonatal Abstinence Syndrome
Source: Pediatr Qual Saf. 2022 Nov 10;7(6):e612. doi: 10.1097/pq9.0000000000000612 (PMC9649270; doi:10.1097/pq9.0000000000000612)
Supplement: Supplementary file 7 [file pqs-7-e612-s007.pdf]

SDC, Unit Huddle Checklist for Patients with NAS

☐ Parents Educated after arrival on expected NAS course, importance of Rooming-In

☐ Parents/Room Provided "The Cuddler Checklist" and "My Comfort Care Plan" resource sheets

- ☐ Parental Contact and Backup Caregiver Contact documented on "My Comfort Care Plan"

☐ Review/Mobilize indicated comfort items (e.g. bassinet, rockers, white noise machine, etc.)

☐ Social Work Consulted (review transportation, other support needs)

- ☐ Completed Barriers to Rooming In Checklist

☐ Child Life Consulted

- ☐ Child Life/Volunteer Coordinator provided with list of expected absences or respite schedule

☐ Substance Use Disorder Service consulted <u>if necessary</u> (review with physician if in question)

Discharge Checklist for Patients with NAS

☐ No significant signs of withdrawal (off medication) for 24-48 hours, other than those which can be managed by the caretaker outside the hospital

☐ No other indication for ongoing hospitalization (feeding, comorbidity, etc.)

☐ Safe discharge care plan established, coordinated with CPS if needed

☐ Parent/caretaker education on NAS and routine care emphasizing safe sleep

☐ Primary care follow-up within 48 hours of discharge

☐ Referred to early intervention services

☐ Home nursing visitation referral (if needed)

☐ Hepatitis C testing follow-up plan in exposed infants (if needed)

☐ Developmental/behavioral pediatrician referral placed (if needed)

Checklist Version 3-3-22
