# Supplementary figures and images for: Quality Improvement Targeting Non-pharmacologic Care and As-needed Morphine Improves Outcomes in Neonatal Abstinence Syndrome
Source: Pediatr Qual Saf. 2022 Nov 10;7(6):e612. doi: 10.1097/pq9.0000000000000612 (PMC9649270; doi:10.1097/pq9.0000000000000612)

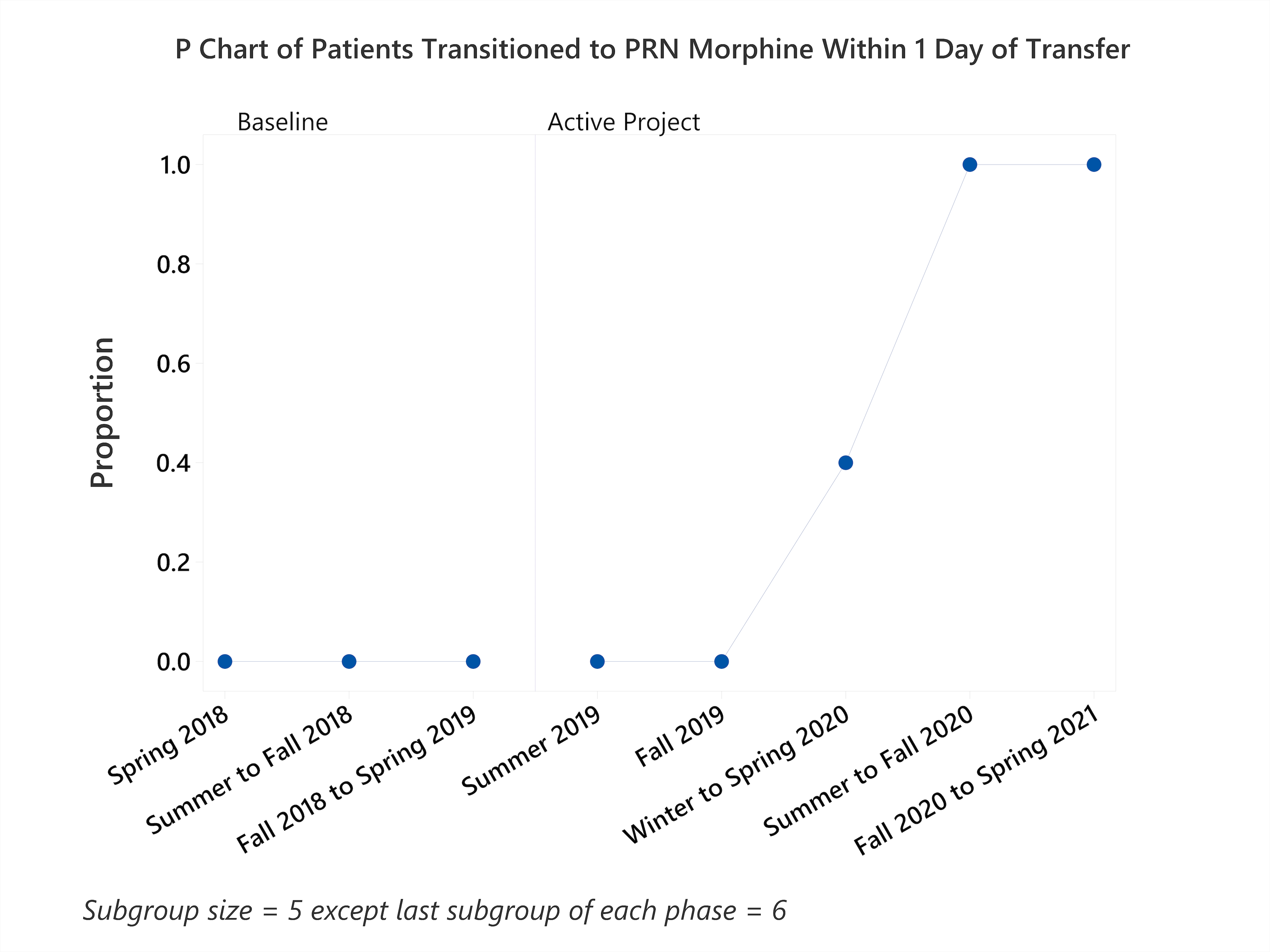

Supplement: Supplementary file 8 [file pqs-7-e612-s008.tif]
